# From barcodes to genomes: a new era of molecular exploration in bryophyte research

**DOI:** 10.3389/fpls.2024.1500607

**Published:** 2025-01-13

**Authors:** Anshul Dhyani, Shruti Kasana, Prem Lal Uniyal

**Affiliations:** Department of Botany, University of Delhi, Delhi, India

**Keywords:** DNA barcoding, hornworts, liverworts, mosses, phylogeny, species delimitation, taxonomy

## Abstract

Bryophytes represent a diverse and species-rich group of plants, characterized by a remarkable array of morphological variations. Due to their significant ecological and economic roles worldwide, accurate identification of bryophyte taxa is crucial. However, the variability in morphological traits often complicates their proper identification and subsequent commercial utilization. DNA barcoding has emerged as a valuable tool for the precise identification of bryophyte taxa, facilitating comparisons at both interspecific and intraspecific levels. Recent research involving plastomes, mitogenomes, and transcriptomes of various bryophyte species has provided insights into molecular changes and gene expression in response to environmental stressors. Advances in molecular phylogenetics have shed light on the origin and evolutionary history of bryophytes, thereby clarifying their phylogenetic relationships. Despite these advancements, a comprehensive understanding of the systematic relationships within bryophytes is still lacking. This review synthesizes current molecular studies that have been instrumental in unraveling the complexity of bryophyte taxonomy and systematics. By highlighting key findings from recent genetic and genomic research, we underscore the importance of integrating molecular data with traditional morphological approaches. Such integration is essential for refining the classification systems of bryophytes and for understanding their adaptive strategies in various ecological niches. Future research should focus on expanding the molecular datasets across underrepresented bryophyte lineages and exploring the functional significance of genetic variations under different environmental conditions. This will not only enhance our knowledge of bryophyte evolution, but also inform conservation strategies and potential applications in biotechnology.

## 1 Introduction

Bryophytes are non-vascular, shade-loving plants characterized by a dominant gametophytic phase (Cox et al., 2014; Liu et al., 2014b; Wickett et al., 2014; Morris et al., 2018; Patiño and Vanderpoorten, 2018). This group includes mosses, hornworts, and liverworts, comprising approximately 20,000 species worldwide (Patiño and Vanderpoorten, 2018). Bryophytes represent the second most diverse group of land plants, surpassed only by flowering plants, and they thrive in a variety of habitats, such as moist, shady, and damp locations, including forest floors, rocks, streams, lakes, and tree trunks (Shaw et al., 2011). Their gametophyte phase exhibits remarkable diversity and structural complexity, which is unparalleled among tracheophytes (Mishler and De Luna, 1991). Bryophytes are distinguished by their unique growth forms, ranging from upright to procumbent, and from thalloid to leafy forms (Mishler and De Luna, 1991).

Interestingly, many bryophytes inhabiting well-illuminated environments function effectively as shade plants, characterized by low chlorophyll a/b ratios and reaching photosynthetic saturation even under low light conditions (Proctor, 1990). Bryophytes also engage in diverse ecological interactions, including obligate symbiosis and occasional epiphytism with a variety of animals and insects (During and Tooren, 1990).

**Figure 1:**
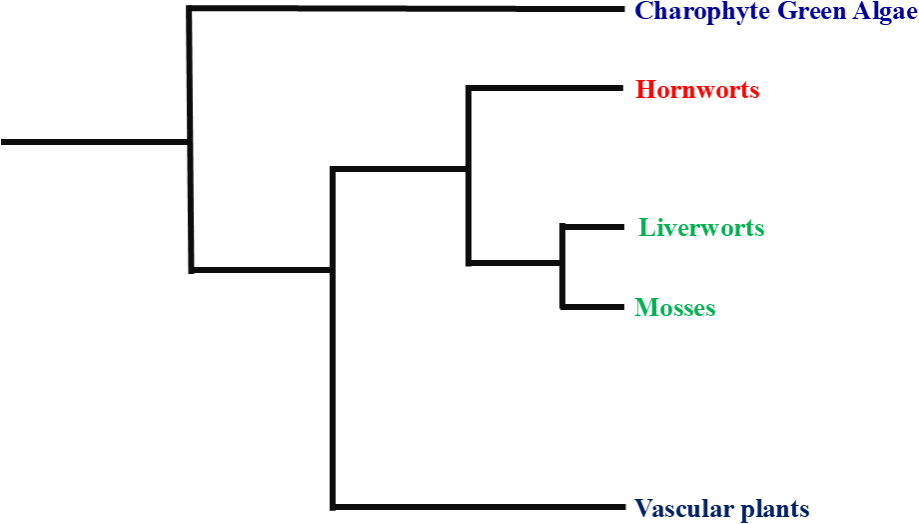
Systematic position of bryophytes in the evolutionary history of land plants.

The phylogenetic position of bryophytes remains a topic of debate. Some studies, such as those by Capesius and Bopp (1997), suggest that bryophytes are paraphyletic, while others, like Nishiyama et al. (2004), argue for their monophyly. These conflicting conclusions highlight the need for more detailed investigations to accurately determine the phylogenetic relationships and evolutionary origins of bryophytes. The phylogenetic tree of bryophytes reveals that liverworts, hornworts, and mosses each occupy distinct evolutionary positions. Charophyte green algae are the most basal group, indicating that land plants evolved from a common ancestor shared with these algae. Hornworts are positioned as an early-diverging lineage among bryophytes, sharing a more recent common ancestor with liverworts and mosses than with green algae. Liverworts and mosses form a clade, suggesting they share a closer evolutionary relationship with each other than with hornworts (**Figure 1**). Continued research utilizing advanced molecular techniques will be essential to resolve these phylogenetic ambiguities and provide a clearer understanding of bryophyte evolution and systematics.

Bryophytes perform a variety of ecological functions and provide essential ecosystem services, including nitrogen and carbon fixation, prevention of soil erosion, water retention, and the maintenance of ecological communities (Ogwu, 2019). Additionally, they have practical applications, such as being used as materials for packaging, plugging, and decoration (Chandra et al., 2017). The chemical compounds found in some bryophyte species exhibit antimicrobial, insecticidal, and antitumor properties, highlighting their potential medicinal value for human health (Üçüncü et al., 2010; Ogwu, 2019). These diverse functional roles underscore the ecological and economic importance of bryophytes and the potential benefits they offer across various sectors.

The classification and grouping of bryophyte taxa based on morphological characteristics face certain limitations due to the high phenotypic plasticity exhibited under varying environmental conditions (Buryová and Shaw, 2005). Environmental factors directly influence morphological traits, leading to significant plasticity, particularly in aquatic mosses (Vanderpoorten and Jacquemart, 2004). This plasticity in stress-tolerant bryophytes is not only morphological but also physiological, with reversible and rapid changes (Grime et al., 1990). From an ecological perspective, morphological plasticity is a key adaptive trait that enhances the fitness of bryophytes by optimizing resource acquisition in variable environments (Rincon and Grime, 1989). However, bryophytes inhabiting unproductive habitats often express plasticity through physiological adjustments rather than morphological changes, focusing on a more conservative strategy of resource capture rather than acquisition (Rincon and Grime, 1989). The relationship between environmental stress and bryophyte genomes has also been explored, revealing that phenotypic plasticity, particularly in response to metal stress, is associated with the presence of highly repetitive DNA sequences (Bassi et al., 2006). These findings underscore the complex interplay between environmental conditions and the genetic mechanisms that underpin the adaptive strategies of bryophytes. Understanding these dynamics is crucial for refining bryophyte classification and for appreciating their ecological roles and adaptive capacities.

Research on the ecology and diversity of bryophytes is often hindered by challenges in species identification and the circumscription of taxa based on traditional taxonomic characters (Stech et al., 2013). Incorrect identification of bryophytes can lead to ambiguous and misleading information in ecological studies, biodiversity assessments, and conservation programs, causing confusion among the scientific community and readers. To address these issues, an integrative approach that combines classical taxonomy with modern molecular techniques is necessary for accurate species identification and delineation. The application of molecular tools, such as DNA barcoding, has proven invaluable for the precise identification of bryophyte species (Dantas et al., 2018). The growing availability of DNA sequences allows for the testing of morphology-based taxonomic concepts by revealing the underlying genotypes of species (Heinrichs et al., 2009). Molecular studies not only facilitate accurate species identification, but also enable detailed exploration of species interactions, both intra- and interspecific. This, in turn, can unravel ecological and evolutionary mechanisms that warrant advanced and in-depth investigation.

Despite high bryophyte diversity, studies on molecular biology of bryophytes from India are meagre. Therefore, in this review, we made an attempt to include some of the barcodes used on Indian bryophytes. We also provide a comprehensive overview of the molecular studies conducted on bryophytes to date, with a particular focus on recent advancements. By highlighting these ecologically significant non-vascular plants, we aim to underscore the importance of molecular approaches in enhancing our understanding of bryophyte diversity, evolution, and ecological roles.

## 2 DNA extraction

Bryophyte samples are typically collected in appropriately sized sampling bags and air-dried to prevent fungal contamination. Careful separation of samples under a dissecting microscope is essential to avoid mixing different taxa. After collection, samples are washed to remove soil and other contaminants that may interfere with subsequent DNA extraction and are then dried. The most commonly used method for extracting genomic DNA from bryophytes is the Cetyl Trimethylammonium Bromide (CTAB) method, as described by Doyle and Doyle (1987). This method has been effectively applied to bryophytes, yielding sufficient DNA for downstream applications such as Polymerase Chain Reaction (PCR). However, bryophytes contain substantial amounts of polysaccharides, polyphenols, and RNA, which can interfere with DNA extraction and PCR amplification (Pandey et al., 2019).

While the CTAB method is widely used, it is both time-consuming and costly (Rogers and Bendich, 1985; Couch and Fritz, 1990; Jobes et al., 1995). Residual CTAB in the DNA solution can also obscure absorbance readings at 260 nm, complicating DNA quantification (Jobes et al., 1995). PCR efficiency may be reduced when extracting genomic DNA from limited plant tissue. Modifications to the CTAB protocol, including optimization with 3% CTAB, 2% Polyvinylpolypyrrolidone (PVP), and 1% β-mercaptoethanol, have improved DNA purity and yield, achieving concentrations of 900–1582 µg per 0.5 g leaf sample in eight bryophyte species (Pandey et al., 2019). Other methods such as the Sodium Dodecyl Sulphate (SDS) (Edwards et al., 1991) and the alkaline isolation of DNA method by Rogers and Parkes (1999) that produced effective DNA concentrations, could be a viable alternative to the CTAB method. These methods, however, also require significant quantities of chemicals. To address these issues, the NaOH extraction and direct amplification method were developed (Werner et al., 2002). These rapid DNA extraction methods are particularly beneficial when processing large numbers of plant samples for studies such as DNA barcoding and molecular phylogenetics. Pederson et al. (2006). employed a rapid DNA extraction method on several bryophytes and successfully obtained sequences from nine moss species and one liverwort species using direct amplification from dwarf male gametophytes. This suggests that rapid extraction methods may serve as suitable alternatives to conventional CTAB protocols. However, comparative studies are needed to evaluate the effectiveness of these methods.

Commercial DNA extraction kits, such as the DNeasy Plant Mini Kit (Qiagen) and the Invisorb Spin Plant Mini Kit (Invitek), have also been used for bryophyte DNA extraction. Among these, the Invisorb Spin Plant Mini Kit was found to yield superior results for AFLP analysis compared to the CTAB method and the DNeasy Plant Mini Kit (Mikulášková et al., 2012). In summary, numerous protocols for bryophyte DNA extraction exist, and these continue to be refined to enhance DNA yield and purity. The choice of method may depend on specific study requirements, such as the desired DNA quality, quantity, and time constraints.

## 3 DNA barcoding

DNA barcoding has become a crucial tool in biodiversity assessment, life history studies, and forensic analysis (Kress et al., 2005). Among the various genetic markers available for the identification and differentiation of land plant species, the internal transcribed spacer 2 (ITS2) region (nuclear) has shown the greatest potential (Chen et al., 2010). In addition to ITS2, chloroplast markers have also been widely employed for DNA barcoding of land plants. However, despite the high species diversity of bryophytes worldwide, DNA barcoding studies in this group have been relatively neglected. This neglect may be attributed to a lack of specialized expertise in bryophyte studies, as well as the small size and less visually appealing nature of bryophytes compared to more conspicuous groups like flowering plants and ferns, which are often larger, brightly colored, and ornamental.

Studies utilizing DNA barcoding in bryophytes with chloroplast and nuclear markers (ITS) have identified several potential candidate regions for species identification, including *rbc*L (plastid), *trn*H-*psb*A (plastid), *rps*4 (plastid), *rpo*C1 (plastid), and *trn*L-F (plastid) (Liu et al., 2010). However, some plastid markers have demonstrated low resolution and poor discriminatory power in mosses, indicating a need for further investigation (Stech and Frey, 2008; Liu et al., 2010; Hassel et al., 2013). For example, *mat*K and *rbc*L show low discriminatory power in some land plants including bryophyta (Stech and Frey, 2008). In contrast, an evaluation of four chloroplast regions (*rbc*L-a, *trn*H-*psb*A, *rps*4, and *trn*L intron) in four genera of the Grimmiaceae suggested that *trn*H-*psb*A can serve as a promising DNA barcode marker for this group (Liu et al., 2011). Further analysis of a nuclear marker (ITS1-5.8S-ITS2) and three plastid markers (*rbc*L, *trn*H-*psb*A, and *mat*K) in the liverwort genus *Herbertus* (Marchantiopsida, Herbertaceae) revealed that ITS has the highest potential for species discrimination, followed by *mat*K (Bell et al., 2012). Another study utilizing three plastid markers (*rbc*L, *trn*H-*psb*A, and *atp*F-H) and one nuclear marker (ITS2) across five moss species and a liverwort species indicated that the most promising barcode markers for bryophytes are ITS2, *rbc*L, and *atp*F-H (Hassel et al., 2013). These findings highlight the necessity of choosing suitable molecular markers for successful DNA barcoding of bryophytes. The continued refinement and advancement of these molecular techniques will be crucial for deepening our understanding of bryophyte diversity and enhancing species identification, especially in ecology and conservation.

The moss *Racomitrium canescens* exhibits considerable morphological variation, leading to differing views regarding its intraspecific taxa and the number of species it comprises. DNA barcoding data indicated that this moss complex cannot be separated into distinct sub-sections or sections. Among the four markers tested (*rps*4-*trn*T, *trn*T-L, ITS1, ITS2), the nuclear marker, ITS1 was identified as the most promising marker due to its superior discriminatory power (Stech et al., 2013). In the genus *Dicranum*, six barcode markers (*rps*4-*trn*T_UGU_, *trn*L_UAA_-*trn*F_GAA_, *trn*H_GUG_-*psb*A, *rps*19-*rp*l2, *rpo*B, and ITS1-5.8S-ITS2) were evaluated for their ability to discriminate between species, with ITS1 emerging as the most effective marker for mosses, particularly for closely related species (Lang et al., 2014). DNA barcoding studies on the family Sematophyllaceae identified four barcode markers (*trn*L-F, *nad*4/5, *rps*4, and *nad*5) as highly effective for taxonomic differentiation (Carvalho-Silva et al., 2014). In the genus *Schistidium*, four DNA barcode markers were assessed, with ITS2 proving to be the most promising due to its high variability and its ability to produce the most resolved phylogenetic tree, followed by *mat*K (Hofbauer et al., 2016).

**Figure 2:**
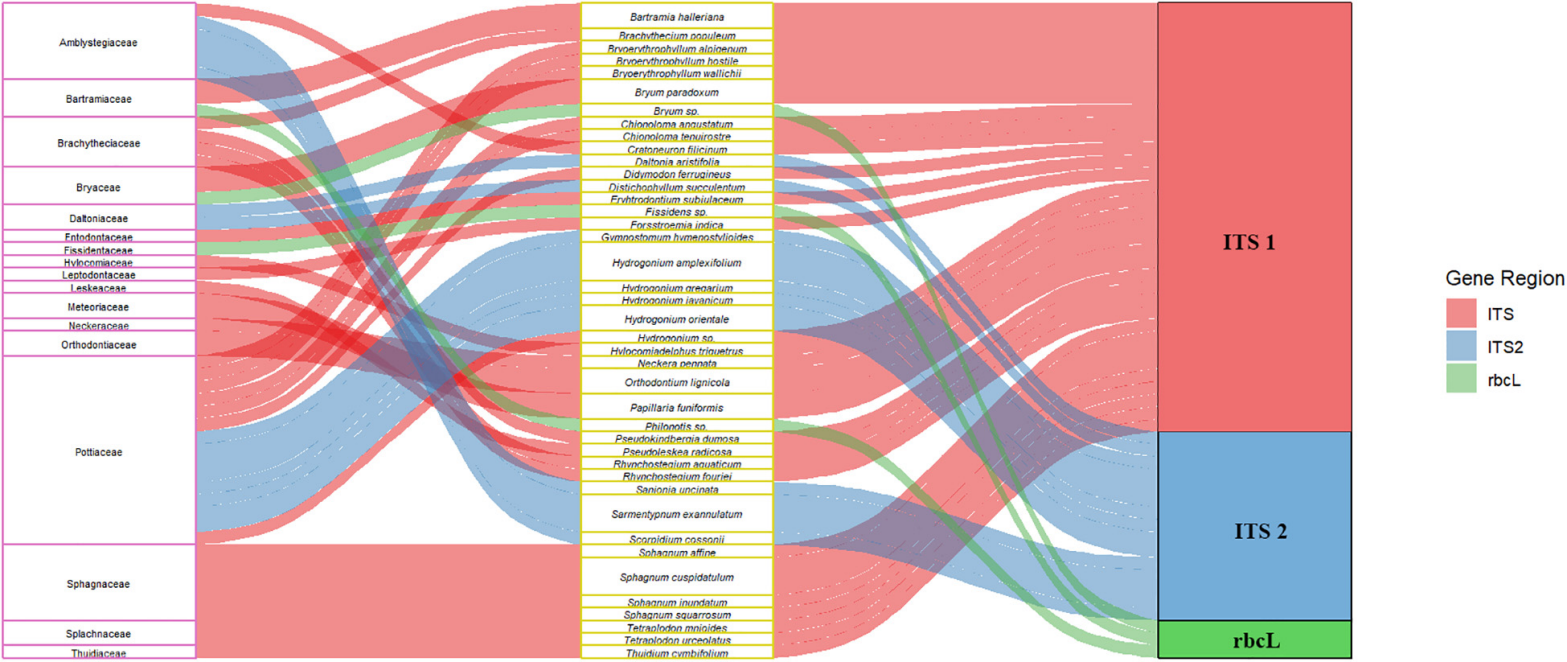
Figure showing the most promising barcodes (ITS1, ITS2, and *rbc*L) available for Indian bryophyte taxa. The first and second columns show the family name and taxa names, and the third column shows the best amplified gene region.

DNA barcoding studies on sixteen species of epiphyllous liverworts using six DNA markers (*trn*L-F, *mat*K, *rbc*L, *psb*A, ITS1, and ITS2) revealed that amplification success ranged from 70% to 90% for all markers except *mat*K. The barcoding gap was found to be highest with ITS2, suggesting it as a promising marker for epiphyllous liverworts (Yodphaka et al., 2018). A recent study on *Calypogeia* tested the plastid genome as a super-barcode for species delimitation and found it to be 95.45% effective. However, complete plastome sequences identified species-specific regions such as *ndh*B, *ndh*H, and the *trn*T-*trn*L spacer, which achieved 100% success in species discrimination across all studied samples (Ślipiko et al., 2020). Despite these advancements, the search for a novel DNA barcode marker that can efficiently differentiate among bryophyte species continues. In India, barcodes are currently available for only 41 species of bryophytes (**Figure 2**) (See also **Supplementary Table S1**).

## 4 RNA editing

RNA editing is a prevalent mechanism observed in some angiosperms and ferns, playing a critical role in correcting genetic information in mitochondrial and chloroplast transcripts. This process involves site-specific conversion of pyrimidine nucleotides, changing C-to-U or U-to-C (Chateigner-Boutin and Small, 2010). While RNA editing occurs in most land plants, the editing pattern of a specific transcript does not necessarily correlate with the species’ phylogenetic position (Freyer et al., 1997). The frequency of RNA editing across land plants is influenced more by lineage than by individual genes (Rüdinger et al., 2009). However, research on RNA editing in bryophytes remains limited. There are numerous potential RNA editing sites identified in the organelle transcriptomes of hornworts, ferns, and seed plants (Kugita et al., 2003; Wolf et al., 2004; Rüdinger et al., 2009; Sloan et al., 2010; Rüdinger et al., 2011).

RNA editing is generally observed in most land plant clades, with notable exceptions among thalloid liverworts (Freyer et al., 1997; Rüdinger et al., 2009). In *Lejeunea cavifolia*, RNA editing is minimal, whereas up to 20% of RNA editing is observed in *Haplomitrium mnioides* (Rüdinger et al., 2012). Among liverworts, Haplomitriales exhibit the highest RNA editing frequency (% RNA editing sites), followed by Pellidae and Metzgeriidae. Rüdinger et al. (2012). analyzed RNA editing patterns in mitochondrial *nad*2, *nad*4, and *nad*5 genes across liverworts and mosses, revealing extensive variability in RNA editing frequencies within both groups. Among leafy liverworts, the order Porellales has the fewest editing sites. The findings indicate that elevated levels of RNA editing may be an ancestral trait in land plants. This high level of RNA editing can be positively correlated with the GC content and diversity of Pentatricopeptide Repeat (PPR) proteins (Takenaka et al., 2008; Takenaka et al., 2013; Dong et al., 2019; Dong and Liu, 2021). However, the intricate patterns and genetic factors involved suggest a complex evolutionary path, with the loss of RNA editing events being more prevalent than their gain. The study also highlights the conserved role of DYW domains in RNA editing, though their exact functions remain elusive. The initial report on RNA editing in the model moss *Physcomitrium patens* indicated a relatively low level of RNA editing, with approximately 20% of *rps*4 sites edited (Miyata and Sugita, 2004). Detailed studies have focused on C-to-U RNA editing in *P*. *patens*, which converts ACG to AUG in the chloroplast *rps*4 transcript—a feature unique to this moss and absent in other species (Barkan and Small, 2014; Ichinose et al., 2014; Gerke et al., 2020). In *P*. *patens*, RNA editing is stage- and tissue-specific (Miyata and Sugita, 2004). These C-to-U or U-to-C conversions can alter coding sequences of organellar transcripts, sometimes correcting premature stop codons or creating start sites like AUG, thus impacting mRNA translation (Small et al., 2020). Despite identifying eleven potential RNA editing sites in *P*. *patens*, only two sites were partially edited in mitochondrial transcripts, highlighting the overall low RNA editing level in this moss (Rüdinger et al., 2009). This low level of RNA editing is potentially correlated with its ten PPR-DYW genes in contrast to angiosperms where *ca.* 100 PPR-DYW genes are present (Tasaki and Sugita, 2010). In contrast, the moss *Funaria hygrometrica* lacks three mitochondrial RNA-editing sites present in *P*. *patens*. *F*. *hygrometrica* has nine DYW proteins, compared to ten in *P*. *patens*, with the absence of the 10th DYW protein explaining the lack of two mitochondrial editing sites (Rüdinger et al., 2011). Experimental analysis in *Takakia lepidozioides* revealed that anticodon editing of tRNA occurs before RNA splicing in plastids, suggesting that RNA editing is a prerequisite for the splicing of pre-tRNALeu (Miyata et al., 2008). Comprehensive analysis of the plastid transcriptome in *T*. *lepidozioides* showed a high frequency of RNA editing, which is positively correlated with the monoplastidy of vegetative tissue. This may be due to the small population size of plastids in vegetative cells, leading to frequent mutation fixation and compensation for deleterious mutations through RNA editing (Sadamitsu et al., 2021).

The studies on RNA editing in hornworts revealed contrasting results. For instance, several authors (Malek et al., 1996; Freyer et al., 1997; Steinhauser et al., 1998; Rüdinger et al., 2009) observed C-to-U editing in all land plants, including liverworts and mosses, but no evidence of U-to-C editing was found in the hornworts and tracheophytes. Contrary to this, other studies (Yoshinaga et al., 1996; Kugita et al., 2003) suggested that reverse RNA editing from U to C is commonly found in the *rbc*L transcript of hornwort chloroplasts. Likewise, RNA editing was found to be extensive in both organelles of *Anthoceros agrestis*, with over 1,100 C-to-U and 1,300 U-to-C sites (Gerke et al., 2020). Similarly, the complete nucleotide sequence of the chloroplast genome of *A*. *formosae* identified 509 C-to-U and 433 U-to-C conversions (Kugita et al., 2003). Moreover, cDNA analysis of seven taxonomically diverse hornworts *rbc*L sequences identified a total of 72 edited sites, comprising 43 C-to-U and 28 U-to-C conversions, with one site showing editing in both directions. All tested samples exhibited extensive RNA editing, except for *Leiosporoceros*, which lacked editing sites. The absence of edited sites in *Leiosporoceros* might be due to the absence or low level of editing in the common ancestor of hornworts (Duff and Moore, 2005). In *Leiosporoceros*, the total number of edited sites was 109 in the plastome and 108 in the mitogenome, corresponding to 0.06% and 0.05%, respectively, further supporting Duff and Moore’s findings (Villarreal et al., 2018). Thus, unlike in mosses and liverworts, RNA editing is highly variable in hornworts, with early-branching lineages tending to have lower RNA editing frequencies (Small et al., 2020). Hence, further research on RNA editing in bryophytes is crucial to unravel the mechanisms underlying its variability across taxa. The findings highlight contrasting patterns, with hornworts exhibiting both C-to-U and U-to-C editing, unlike the tracheophytes, which lacks U-to-C editing. Extensive RNA editing in organelles of species like *A*. *agrestis* contrasts with the absence of editing in *Leiosporoceros*, suggesting ancestral differences, emphasizing the need for more studies to understand these evolutionary trends.

## 5 Transcriptome analysis

Transcriptome analysis enables a detailed study of the expression profiles of thousands of genes, and numerous molecular studies on bryophytes have employed this approach. Most transcriptome studies in bryophytes focus on the stress adaptation mechanisms present in these plants. Analyzing mRNA levels in a cell provides more valuable insights into molecular changes than measuring the amount of protein encoded by genes (Woll et al., 2005). Above all, transcriptomics is comparatively easier than proteomic studies. Transcriptome analysis is crucial for understanding gene expression profiles under various conditions and detecting molecular changes within the cell.

The first *de novo* transcriptome analysis of male and female gametophyte assemblies in *Marchantia polymorpha* generated 80 million sequence reads and identified several new transcription factors (TFs) families such as GRAS, LEAFY, NOZZLE, LUG, etc. that play important role in sexual reproduction (Sharma et al., 2013). Transcriptomic studies on *P*. *patens* revealed the accumulation of several late-embryogenesis-abundant (LEA) transcripts in response to enhanced freezing tolerance under both light and dark conditions (Minami et al., 2005).

Transcriptome sequencing of *Dumortiera hirsuta* resulted in 85,240 unigenes and 447 TFs from 41 different families. These unigenes showed homology across different taxa from algae to flowering plants which could be seen as a potential connecting link between aquatic and terrestrial plants (Singh et al., 2015). Expression profile studies on *Marchantia inflexa* under water stress revealed changes in transcripts related to metabolism. These studies suggest that variations in the timing of transcript adjustments contribute to differences among genotypes, impacting stress tolerance in both meristematic and differentiated cells (Marks et al., 2021). In *Mylia taylorii*, transcriptome analysis identified that the enzyme sesquiterpene synthase (STS) plays a role in sesquiterpene biosynthesis and diversity (Yan et al., 2021). The liverwort transcriptome contained 255,669 unigenes with an average length of 963 base pairs, with 48 unigenes potentially involved in sesquiterpene biosynthesis. Functional characterization in yeast indicated that MtSTSs exhibit a noncanonical metal ion binding motif, highlighting their contribution to sesquiterpene biosynthesis and the biological roles of these compounds in *M. taylorii*.

*Bryum argenteum*, a desiccation-tolerant moss, is increasingly being used as a model to study the ecological and molecular aspects of desiccation tolerance in plants (Stark et al., 2010; Li et al., 2014; Gao et al., 2015). Transcriptome analysis of *B*. *argenteum* using Illumina high-throughput RNA sequencing technology generated more than 488.46 million reads (Gao et al., 2017). Annotation of TFs revealed that 978 TFs belong to 62 families, with 404 TFs from 40 families showing differential expression upon dehydration followed by rehydration (Gao et al., 2017). In this moss, mRNA transcripts accumulate in messenger ribonucleoprotein particles (mRNPs) in response to desiccation. Upon rehydration, these transcripts are selectively translated through the activation of repair-based mechanisms (Gao et al., 2017). In *Tortula ruralis*, another desiccation-tolerant moss, the pattern of protein formation during rehydration differs from that of hydrated controls, as novel transcripts are not synthesized due to desiccation (Oliver et al., 2004). This indicates that desiccation tolerance mechanisms can vary significantly even among different moss species, highlighting the importance of comparative studies.

Transcriptome studies have also been conducted on the Antarctic moss *Pohlia nutans* to understand its response to salt stress. A common strategy under high salt stress involves the activation and regulation of downstream proteins required for cell repair and adaptation, including those related to ion homeostasis, osmoregulation, and reactive oxygen species (ROS) scavenging (Hu et al., 2018; Zhang et al., 2019). In response to salt stress, *P*. *nutans* exhibited upregulation of 1,340 genes and downregulation of 831 genes. Additionally, this moss activated various phytohormone signaling pathways that stimulate antioxidant enzymes and flavonoids to protect cells and scavenge ROS (Zhang et al., 2019). In the hornwort *Folioceros fuciformis*, transcriptome sequencing has revealed the presence of homologs of Dicer-Like (DCL), Argonaute (AGO), and various other genes involved in small RNA pathways (You et al., 2017). However, the literature on transcriptome studies in hornworts is limited, indicating a need for further research to better understand the molecular changes and gene expression dynamics in this group of bryophytes.

## 6 Plastome analysis

**Figure 3:**
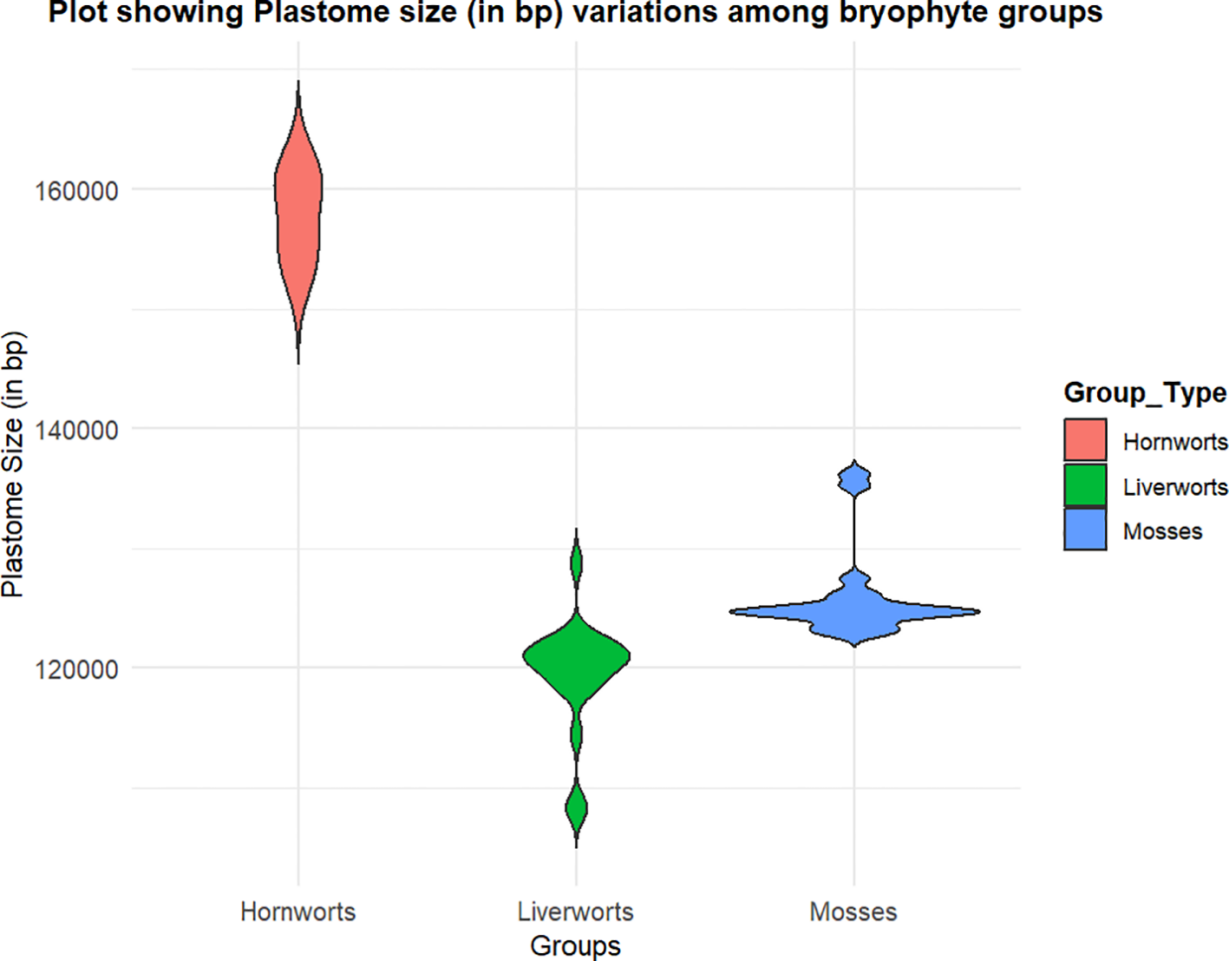
Violin plot showing plastome size in base pair (bp) among three groups of Bryophytes: Mosses (blue), Liverworts (green), and Hornworts (peach). The width of each violin represents the density of plastome size distribution within each group. The y-axis indicates plastome size in bp.

The basic structure of the plastome in bryophytes consists of a large single-copy (LSC) region and a small single-copy (SSC) region, flanked by a set of large, inverted repeats. The distribution of plastome sizes (in base pairs) across different bryophyte groups is represented in **Figure 3**. The first complete plastome study in *M*. *polymorpha* revealed that its chloroplast DNA (cpDNA) comprises a total of 121,024 base pairs, with two large, inverted repeats separating the LSC and SSC regions (Ohyama et al., 1986). This plastome contains 128 genes, which include ribosomal RNAs, 32 species of transfer RNAs (tRNA), and 55 identified open reading frames (ORFs) for proteins (Ohyama et al., 1986). Since then, the plastome of many bryophytes has been sequenced (**Supplementary Table S2**).

In *Aneura mirabilis*, a total of 20 pseudogenes were identified based on complete chloroplast genome sequence, which include the loss of five chlororespiration genes (*ndh*), six other *ndh* genes, subunits of photosystem I and II, cytochrome *b6f* complex, and also involves the pseudogenes of *ccs*A, *cyst*, *cys*A, and *ycf*3 (Wickett et al., 2008; Wolf and Karol, 2012). In the hornwort *Nothoceros aenigmaticus*, the plastid genome was reconstructed using a shotgun sequencing approach of genomic DNA. The plastome of *Nothoceros* was found to be collinear with the plastomes of other bryophytes but differed from the *Anthoceros* plastome in many gene regions. These differences are particularly evident within the inverted repeat regions of *Anthoceros* (Villarreal et al., 2013). This body of research underscores the diversity in plastome organization among different bryophytes, providing insights into the evolutionary adaptations of their chloroplast genomes. In the plastome sequence of the moss *P*. *patens*, Sugiura et al. (2003) identified 83 protein-coding genes, four ribosomal RNA genes, 31 tRNA genes, and a pseudogene. Notably, four genes— *rpo*A, cysA, *cys*T, and *ccs*A— present in *M*. *polymorpha* and *A*. *formosae* were absent in *P. patens*. Additionally, the overall structure of the cpDNA in *P. patens* differs from that of *M. polymorpha* and *A. formosae*. Specifically, a large inversion is unique to *P. patens*, while the loss of the *rpo*A gene is a common feature observed across all studied mosses (Sugiura et al., 2003).

In the moss *Sanionia uncinata*, the total plastome length is 124,374 bp with a total of 117 unique genes which comprises 82 protein-coding genes, 37 tRNA genes and four genes for rRNA (Park et al., 2018). Plastome analysis in moss *T*. *ruralis* showed that the chloroplast genome is ca. 123,500 bp, and it was different from that of *P. patens* in the sense that *Tortula* does not have the ~71 kb inversion found in the LSC region of the *Physcomitrella* genome (Oliver et al., 2010). In *Polytrichum commune*, the plastome is 126,323 bp in length and comprises four regions: one LSC region of 88,070 bp, a SSC region of 16,717 bp, and two inverted repeats (IRs) of 9,680 bp each (Jin and Zhu, 2021). It contains a total of 128 genes, including 84 protein-coding genes, eight ribosomal RNA (rRNA) genes, and 36 transfer RNA (tRNA) genes. Also, in *P.* commune nine genes (four rRNA genes and five tRNA genes) are duplicated in the IR regions (Jin and Zhu, 2021).

Significant differences in plastome structure have been observed among liverworts. Yu et al. (2019) examined these variations, focusing on genome size and GC content, and found that *Aneura* and *Haplomitrium* exhibit higher GC content compared to other liverworts, showing a 1.54-fold variation in GC content across the studied liverworts. When comparing the plastomes of 2,386 land plants, they noted structural conservatism in liverwort plastomes, with a trend towards reduced plastome length from liverworts and mosses to land plants. Among mosses, *Takakia* and *Sphagnum* have plastome sizes comparable to those of land plants. However, the disparity in plastome structure among hornworts remains unexplored, and a detailed study is needed to gain further insights into their evolutionary trends.

## 7 Genetic diversity

The exploration of genetic diversity in bryophytes started to develop in the early 2000s. Cronberg (2000) examined genetic variation in *Leucodon sciuroides* by analyzing 15 putative isozyme loci across twelve populations. This study found that Scandinavian populations had lower genetic diversity compared to Greek populations, with northern Greece acting as a transitional area between genetically impoverished and diverse populations. This pattern suggests that genetic variation diminished in populations located at the northern limits of glacial refugia. Furthermore, the study highlighted differences in reproductive strategies among populations, suggesting that epiphytic species, which are limited in space and time, are more vulnerable to genetic variation loss (Cronberg, 2000).

In a separate study, Cronberg et al. (2005) investigated genetic variation in *Plagiomnium affine* by examining 23 allozyme loci across six populations. They identified sixteen haplotypes, with two being widely distributed and twelve unique to specific populations. The study revealed a significant correlation between allelic variation and forest age, with dominant haplotypes found in younger forests and local haplotypes more prevalent in older forests. This indicates that genetic variation tends to accumulate more in ageing forest ecosystems. Using the random amplified polymorphic DNA (RAPD) technique, Zhu et al. (2007) analyzed 60 individuals from five Chinese populations of *Brachythecium rivulare*. They identified a total of 122 bands, with 82 (67.2%) showing polymorphism, indicating a substantial level of genetic variation. However, no significant correlation was found between genetic distance and elevational gradient among these populations. In contrast, a genetic diversity study of *Bryum argenteum* collected from elevations ranging from 100 m to 2870 m revealed that genetic diversity peaked at 1900 m. This study found a significant correlation between genetic variation and elevation, but no correlation between genetic variation and geographic distance, with no demographic shifts observed at any elevation (Pisa et al., 2013). Among the four bryophytes examined (*Exsertotheca intermedia*, *Frullania polysticta*, *Isothecium prolixum*, and *Porella canariensis*), a correlation between species richness and genetic diversity was observed in *I*. *prolixum* and *E*. *intermedia*, which showed higher species cover and genetic diversity at higher elevations (Sim-Sim et al., 2015).

Hock et al. (2008) examined the genetic diversity in two populations of *Mannia fragrans*— one from the soil surface and the other from the diaspore bank using three Inter-Simple Sequence Repeats (ISSR) primers. They found that genetic diversity was similar in both populations (0.067 for soil surface and 0.082 for diaspore bank). However, specific haplotypes were unique to the soil surface population, highlighting the significant role of the bryophyte diaspore bank in preserving genetic variability across generations. Lang et al. (2021) investigated the genetic structure and variability of sexually reproducing populations of *Dicranum scoparium* at different geographic levels, focusing on the relative contributions of dwarf males (DMs), females, and normal-sized males (NMs) to genetic diversity. Using 119 single nucleotide polymorphism (SNP) markers from transcriptomes to genotype 403 samples, they observed that DMs, when present, significantly outnumbered NMs and females at certain sites. Local-level genetic differentiation was low, but significant differentiation was noted between cushions for NMs and females and within cushions for DMs. While genetic diversity was lower for NMs, it was comparable between females and DMs. The study revealed that DMs and NMs play distinct roles in reproduction, with inbreeding potential at the cushion level but high gene flow preventing substantial genetic drift (Lang et al., 2021). These findings underscore that genetic diversity in bryophytes is influenced not only by genetic and genomic factors but also by ecological and reproductive dynamics. An integrative approach considering these aspects could further elucidate the mechanisms underlying genetic diversity and its connections with broader biological contexts.

## 8 Molecular phylogeny

Identifying and classifying bryophytes solely based on morphology can be challenging due to their small size, diverse growth conditions, and phenotypic plasticity. Therefore, an integrative approach combining various methods is essential for accurate classification. Molecular systematics, which relies on phylogenetic reconstruction, is a widely used approach for classifying bryophytes. Bryophytes are considered the original colonizers of terrestrial habitats, and their status as some of the oldest living land plants is rarely disputed (Mishler and Churchill, 1984). Molecular markers have long been employed in the phylogenetic analysis of bryophytes and other land plants and remain essential tool to understand phylogenetic relationships. Commonly used markers include the nuclear 18S RNA and the chloroplast *rbc*L gene (Beckert et al., 1999). Additionally, the Internal Transcribed Spacer (ITS1-4) region from the nuclear genome has been used to infer bryophyte phylogeny at the molecular level (Samigullin et al., 1998). Results from molecular phylogenetic studies of bryophytes have been controversial. Early views suggested that bryophytes represented a grade consisting of three monophyletic lineages with no clear branching order (Mishler et al., 1994). Studies of nuclear-encoded rRNA genes proposed that the hornwort-moss clade is sister to tracheophytes, with liverworts positioned as basal to tracheophytes, although the moss-hornwort clade was weakly supported (Waters et al., 1992). Concatenated nucleotide data analyses suggest mosses as the sister group to all other land plants, while corresponding amino acid sequences position liverworts as the sister group to land plants (Liu et al., 2014a). Ruhfel et al. (2014) demonstrated a strong relationship between mosses and liverworts, forming a distinct clade. However, this clade was found to be distantly related to hornworts and other embryophytes, with no evidence supporting the monophyly of bryophytes. In contrast, Gitzendanner et al. (2018) provided strong evidence for bryophyte monophyly, placing hornworts as sister to a moss-liverwort clade. Additionally, phylogenomic studies of the hornwort *Anthoceros angustus* also suggested the monophyly of bryophytes, with hornworts considered sister to liverworts and mosses (Zhang et al., 2020). Study on the mitochondrial phylogeny corroborated the Setaphyta clade, reinforcing the concept of bryophyte monophyly with robust support (Sousa et al., 2020). Phylogenetic analysis based on nuclear proteins further supported the monophyly of bryophytes (de Sousa et al., 2019). Also, study by Su et al. (2021) based on large-scale phylogenomic analysis further advocate the monophyly of bryophytes. Likewise, phylogenetic analysis using translated amino acid sequences from chloroplast genomes of twenty bryophyte species strongly supports the monophyly of extant bryophytes as sister to vascular plants, though the support for the monophyly of vascular plants was weaker (Nishiyama et al., 2004). Contrary to this, analysis of the nuclear 18S RNA gene sequence, using parsimony and maximum likelihood methods, has placed hornworts as a basal group, with mosses and liverworts forming sister taxa to each other and together constituting a sister clade to the tracheophytes (Hedderson et al., 1996). Mitochondrial *nad*5 gene sequences revealed the monophyly of mosses, liverworts, and hornworts, with *nad*5-derived phylogenetic trees supporting some taxonomic units in bryophyte classification (Beckert et al., 1999). In contrast, Wickett et al (2014). argued that bryophytes are paraphyletic, with liverworts and mosses being sister to vascular plants, while hornworts are sister to all other land plants. As aforementioned, phylogenomic analyses have supported bryophytes as monophyletic, with hornworts being sister to the Setaphyta clade, which includes both liverworts and mosses (Wang et al., 2022).

**Figure 4:**
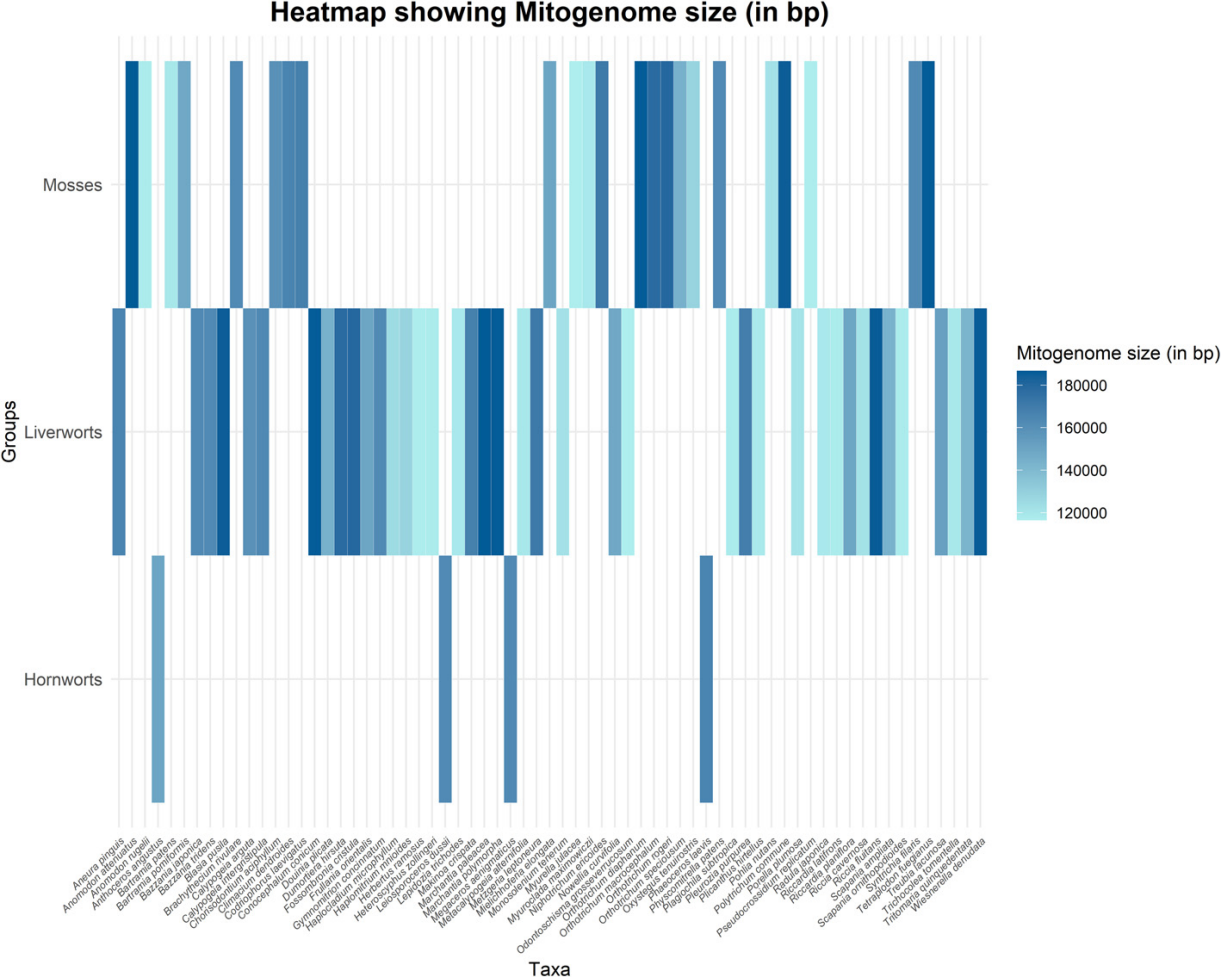
Heatmap illustrating mitogenome size (in bp) across various bryophyte taxa. The X-axis represents individual taxa, while the Y-axis categorizes them into three bryophyte groups: Mosses, Liverworts, and Hornworts. The color gradient corresponds to mitogenome size, with darker shades indicating larger mitogenomes and lighter shades representing smaller ones.

A recent study focused on liverworts, specifically the order Ptilidiales, using 84 protein-coding genes from the chloroplast genome, supported the monophyly of liverworts, with Ptilidiales identified as sister to Jungermanniales (Yu et al., 2020). In mosses, a study on *P*. *commune* using 33 mitochondrial coding genes revealed that its mitogenome is highly similar to other Polytrichopsida members, with the least similarity to *Buxbaumia aphylla* and *Sphagnum palustre* (Goryunov et al., 2021). The mitogenome of studied bryophytes is also represented in **Figure 4** (See also **Supplementary Table S3**). In the family Funariaceae (Bryophyta), the phylogenetic analysis did not support the classification based solely on sporophyte morphology, suggesting that sporophyte characteristics are homoplastic and that selective pressures have led to diversification in sporophytic architecture (Liu et al., 2012). Additionally, research on the evolution of stomata has supported the monophyly of bryophytes (Harris et al., 2020). Conversely, a study on haplolepidous moss families Aongstroemiaceae and Dicranellaceae, using chloroplast and mitochondrial markers, revealed that these morphologically similar families are actually separate clades and polyphyletic (Bonfim Santos et al., 2021). Continued research is needed to resolve the phylogenetic relationships of the complex bryophyte families to achieve a more precise classification.

In liverworts, Capesius and Bopp (1997) supported them as paraphyletic, linking simple thalloid and leafy species to mosses while excluding complex thalloid liverworts. At family level, the family Lejeuneaceae is the most species-rich, but its classification has been challenging due to significant morphological homoplasy. This has led to conflicting classifications and difficulties in dividing the family into natural subunits (Wilson et al., 2007). Phylogenetic analyses using plastid regions (*rbc*L, *trn*L-F, and *psb*A) and the nr5.8S-ITS2 region identified four main lineages within Lejeuneaceae supporting this division. The study further suggested separating Lejeuneaceae into the subfamilies Ptychanthoideae and Lejeuneoideae (Hentschel et al., 2009). The genus *Frullania* within the family Frullaniaceae of order Porellales is also taxonomically complex. Morphologically, *Frullania* is similar to Jubulaceae and Lejeuneaceae, leading some classifications to group *Frullania*, *Jubula*, and *Lejeunea* within a single family, Jubulaceae (Hentschel et al., 2009). However, Schuster (1994) and Crandall-Stotler and Stotler (2009) proposed separating *Frullania* and *Jubula* from Lejeuneaceae, placing them solely in Jubulaceae. Phylogenetic analysis of *Frullania* has supported the monophyly of several subgenera and their intercontinental ranges, which contrasts with the morphology-based classification of subgenera (Schuster, 1994).

In the family Lepidoziaceae, which belongs to the order Jungermanniales, there is significant gametophytic polymorphism, leading to challenges in establishing stable taxonomic boundaries at the subfamily, subgeneric, or generic levels (Crandall-Stotler and Stotler, 2009). Despite extensive taxonomic studies, the phylogenetic relationships within Lepidoziaceae remain unclear. Cooper et al. (2011) conducted a comprehensive study using a large dataset of 10 loci and 93 species from 20 genera, providing convincing evidence against the monophyly of the three subfamilies—Lembidioideae, Lepidozioideae, and Zoopsidioideae. Their analysis proposed a revised classification, including a narrower circumscription for Lepidozioideae and reassignments such as *Megalembidium insulanum* to Lembidioideae and *Neogrollea notabilis* to Lepidoziaceae.

The intricate relationships between the moss families Grimmiaceae and Ptychomitriaceae have been a long-standing focus of research due to the challenges in classifying various taxa within these families. Phylogenetic studies have produced differing conclusions on whether these families should be merged or kept separate. For instance, earlier studies such as Noguchi (1987), Gradstein et al. (2001), Allen (2002), and Tsubota et al. (2001), suggested merging both families into one. In contrast, other studies such as Buck et al. (2000) and Smith et al (2004). advocated for maintaining them as separate entities. A more recent study by Hernández-Maqueda et al. (2008) provided new insights by placing the genera *Indusiella* and *Jaffueliobryum* within Ptychomitriaceae due to their close relationship with the genus *Ptychomitrium*. The study also proposed that *Campylostelium* does not fit within either Grimmiaceae or Ptychomitriaceae and should be classified in its own family, Campylosteliaceae. Additionally, they identified *Racomitrium* as a monophyletic group closely related to a clade consisting of *Grimmia*, *Schistidium*, and *Dryptodon*. The study also supported the synonymization of *Coscinodon* with *Grimmia* and established *Grimmia* and *Schistidium* as a monophyletic group with strong statistical support. In another lineage, Sphagnopsida, the genus *Sphagnum*, with around 350−500 species, and *Ambuchanania*, represented by a single species, have been the subject of phylogenetic analysis. Initially placed in a new section of *Sphagnum*, *Ambuchanania* was later assigned to a separate family and order (Crum and Seppelt, 1999; Shaw et al., 2010). Phylogenetic analyses of Sphagnopsida have identified three primary lineages: 1) *S*. *sericeum*, 2) *S*. *inretortum* plus *A*. *leucobryoides*, and 3) all other *Sphagnum* species. This analysis also indicated that *A*. *leucobryoides* is derived within Sphagnopsida rather than being plesiomorphic (Crum and Seppelt, 1999; Shaw et al., 2010).

The phylogenetic relationships within the family Daltoniaceae and the circumscription of this family still require further investigation. Some genera, such as *Calyptrochaeta*, have yet to be definitively placed within the family (Ho et al., 2012). Phylogenetic analyses of Daltoniaceae using five markers from all three genomic regions supported the reciprocal monophyly of *Calyptrochaeta* and *Achrophyllum*. However, genera like *Daltonia*, *Leskeodon*, and *Distichophyllum* were found to be polyphyletic, indicating the need for extensive taxonomic revisions within this family (Ho et al., 2012).

The current state of hornwort taxonomy presents significant challenges, making it difficult to pinpoint the exact number of hornwort species globally. As of now, over 300 hornwort species have been documented worldwide (Duff et al., 2007). Taxonomic classification within hornworts has led to several conflicting concepts of their interrelationships (Cargill et al., 2005; Duff et al., 2007). Early phylogenetic studies on hornworts, such as those by Duff et al. (2007) and Stech et al. (2003) laid the groundwork for understanding their classification. These studies, based on *rbc*L phylogeny, highlighted significant genetic divergence within hornworts, including the distinct separation of *Phaeoceros* and *Anthoceros*, the divergence of *Leiosporoceros* from other hornworts, the polyphyly of *Megaceros*, and the existence of a cryptic genus containing species formerly classified under *Phaeoceros* (Stech et al., 2003; Duff et al., 2007). Newer classifications proposed by Frey & Stech (2005) and Stotler & Crandall-Stotler (2005) exhibit notable similarities but also differ significantly from earlier frameworks. Despite these advances, hornwort biology remains underdeveloped, partly due to the scarcity of specialists in hornwort taxonomy and the challenges of accessing hornwort populations in remote areas.

## 9 Application of molecular studies in bryology

Morphological classification of bryophytes often struggles to accurately group these diverse taxa, revealing just how complex their taxonomy can be. With the advent of the molecular revolution, advances in genetic studies have become essential for addressing these challenges and accurately distinguishing species, particularly within complex species groups (Beckert et al., 1999; Stech et al., 2013; Lang et al., 2015; Duffy et al., 2020). By analyzing nucleotide and amino acid sequences, molecular tools can classify species as monophyletic, paraphyletic, or polyphyletic, complementing traditional morphological approaches (Vanderpoorten and Shaw, 2014). Despite their transformative potential, molecular studies on bryophytes remain underutilized, demanding a prominent level of expertise to tackle classification complexities and refine species delineation. Yet, these detailed molecular analyses promise to unravel intricate biological processes, including cell signaling pathways, which are vital for ecological, molecular, and biodiversity conservation studies. By advancing molecular research, we can enhance overexpression studies and explore new applications, transforming our approach to these remarkable plants. Thus, an integrative approach, blending molecular and morphological methods, is essential not only for resolving species complexes but also for confirming and reclassifying misidentified or enigmatic species.

While molecular research on bryophytes is rapidly gaining momentum globally, India’s vast and diverse bryophyte flora remains underexplored at the molecular level. Despite hosting four biodiversity hotspots and approximately 2,562 bryophyte taxa (ENVIS, India), there is a noticeable lack of molecular studies within the country. This gap can largely be attributed to a shortage of specialists in bryophyte taxonomy and a general lack of awareness regarding the importance of bryophyte taxonomy, ecology, and conservation. To address this issue, several strategic approaches are essential. Government policies focused on bryophyte conservation, along with the organization of targeted training programs and workshops, can play a crucial role in fostering expertise and awareness. Additionally, developing bryophyte gardens would provide valuable resources for research and education, further promoting the study and conservation of these crucial but often overlooked plants. By prioritizing these initiatives, India can enhance its contributions to the global understanding of bryophytes and ensure that its rich bryophyte diversity is effectively studied, conserved, and appreciated.

## 10 Conclusion

Molecular studies have emerged as transformative tools in resolving the complex classification issues that often plague bryophytes. In a field where overlapping morphological characters frequently blur taxonomic boundaries, molecular techniques offer precise methods for species discrimination and classification. An integrative approach that combines molecular data with traditional systematic botany is essential for achieving accurate and comprehensive classifications of bryophytes. Recent advancements in DNA extraction protocols for bryophytes have set new benchmarks, encouraging researchers to explore innovative techniques for extracting and isolating DNA from these delicate plants. Genome-wide studies and transcriptome analyses are shedding light on the intricate genetic frameworks of bryophytes, with ongoing efforts in genome sequencing and assembly providing deeper insights into their evolutionary histories. The field has also benefitted from advances in molecular phylogeny and DNA barcoding, which have proven invaluable for delineating species within challenging groups like Grimmiaceae and Pottiaceae. However, the quest for a universally efficient DNA barcode marker remains a matter of comprehensive investigations. Whether a single marker or a combination of markers will emerge as the definitive tool for bryophyte species identification is still under investigation. Continued research is crucial to establishing a standard DNA barcode marker that can aid bryologists globally, paving the way for more accurate and reliable studies of bryophyte diversity.
