# Supplementary material for: From barcodes to genomes: a new era of molecular exploration in bryophyte research
Source: Front Plant Sci. 2025 Jan 13;15:1500607. doi: 10.3389/fpls.2024.1500607 (PMC11770019; doi:10.3389/fpls.2024.1500607)
Supplement: Supplementary file 1 [file Table1.docx]

**Table S1:** Table shows the Indian taxa for which the barcodes are available along with the gene region used

| **Taxon** | **Family** | **Sample ID** | **Gene region** | **Locality** |
| --- | --- | --- | --- | --- |
| *Bartramia halleriana* | Bartramiaceae | MN294930 | ITS | - |
| *Bartramia halleriana* | Bartramiaceae | MN268741 | ITS | - |
| *Brachythecium populeum* | Brachytheciaceae | MN398589 | ITS | - |
| *Bryoerythrophyllum alpigenum* | Pottiaceae | MN307167 | ITS | - |
| *Bryoerythrophyllum hostile* | Pottiaceae | KY406813 | ITS | Sikkim, India |
| *Bryoerythrophyllum wallichii* | Pottiaceae | KY406811 | ITS | Sikkim, India |
| *Bryum paradoxum* | Bryaceae | MN398588 | ITS | - |
| *Bryum paradoxum* | Bryaceae | MN268742 | ITS | - |
| *Bryum* sp. | Bryaceae | DNAFR000466 | *rbc*L | Gujarat, India |
| *Chionoloma angustatum* | Pottiaceae | KT380445 | ITS | - |
| *Chionoloma tenuirostre* | Pottiaceae | KT380422 | ITS | - |
| *Cratoneuron filicinum* | Amblystegiaceae | MG572784 | ITS | - |
| *Daltonia aristifolia* | Daltoniaceae | GQ905886 | ITS2 | - |
| *Didymodon ferrugineus* | Pottiaceae | MF536560[ | ITS | - |
| *Distichophyllum succulentum* | Daltoniaceae | HQ613525 | ITS2 | West Bengal, India |
| *Eryhtrodontium subjulaceum* | Entodontaceae | MN398587 | ITS | - |
| *Forsstroemia indica* | Leptodontaceae | MN398590 | ITS | - |
| *Gymnostomum hymenostylioides* | Pottiaceae | HM147819 | ITS2 | Uttarakhand, India |
| *Hydrogonium amplexifolium* | Pottiaceae | JQ890504 | ITS2 | Sikkim, India |
| *Hydrogonium amplexifolium* | Pottiaceae | JX679937 | ITS2 | Uttarakhand, India |
| *Hydrogonium amplexifolium* | Pottiaceae | JX679938 | ITS2 | Uttarakhand, India |
| *Hydrogonium gregarium* | Pottiaceae | JQ890487 | ITS2 | Uttarakhand, India |
| *Hydrogonium javanicum* | Pottiaceae | JQ890519 | ITS2 | Sikkim, India |
| *Hydrogonium orientale* | Pottiaceae | JQ890493 | ITS2 | Uttarakhand, India |
| *Hydrogonium orientale* | Pottiaceae | JQ890497 | ITS2 | Lucknow, India |
| *Hydrogonium* sp. | Pottiaceae | MG572787 | ITS | - |
| *Hylocomiadelphus triquetrus* | Hylocomiaceae | MN398586 | ITS | - |
| *Neckera pennata* | Neckeraceae | MN294929 | ITS | - |
| *Orthodontium lignicola* | Orthodontiaceae | MK309722 | ITS | Sikkim, India |
| *Orthodontium lignicola* | Orthodontiaceae | MK309710 | ITS | Sikkim, India |
| *Papillaria funiformis* | Meteoriaceae | MK170396 | ITS | - |
| *Papillaria funiformis* | Meteoriaceae | MK170395[ | ITS | - |
| *Pseudokindbergia dumosa* | Brachytheciaceae | KJ659017 | ITS | Uttarakhand, India |
| *Pseudoleskea radicosa* | Leskeaceae | MN186844 | ITS | - |
| *Rhynchostegium aquaticum* | Brachytheciaceae | MG572788 | ITS | - |
| *Rhynchostegium fouriei* | Brachytheciaceae | MN294928 | ITS | - |
| *Sanionia uncinata* | Amblystegiaceae | HQ452106 | ITS2 | - |
| *Sarmentypnum exannulatum* | Amblystegiaceae | FJ474549 | ITS2 | Sikkim, India |
| **Taxon** | **Family** | **Sample ID** | **Gene region** | **Locality** |
| *Sarmentypnum exannulatum* | Amblystegiaceae | FJ474552 | ITS2 | Sikkim, India |
| *Sarmentypnum exannulatum* | Amblystegiaceae | FJ474554 | ITS2 | Sikkim, India |
| *Scorpidium cossonii* | Amblystegiaceae | EU563668 | ITS2 | Sikkim, India |
| *Sphagnum affine* | Sphagnaceae | AY298362 | ITS | - |
| *Sphagnum cuspidatulum* | Sphagnaceae | AY298428 | ITS | - |
| *Sphagnum cuspidatulum* | Sphagnaceae | AY298426 | ITS | - |
| *Sphagnum cuspidatulum* | Sphagnaceae | AY298425 | ITS | - |
| *Sphagnum inundatum* | Sphagnaceae | AY298508 | ITS | - |
| *Sphagnum squarrosum* | Sphagnaceae | AY298649 | ITS | - |
| *Tetraplodon mnioides* | Splachnaceae | KJ488253 | ITS | - |
| *Tetraplodon urceolatus* | Splachnaceae | KJ488284 | ITS | - |
| *Thuidium cymbifolium* | Thuidiaceae | MN294927 | ITS | - |

**Table S2:** Table showing the list of bryophytes with information about the chloroplast genome

| **S. No.** | **Bryophytes** | **Plastome Size** | **References** |
| --- | --- | --- | --- |
|  | **Liverworts** |  |  |
|  | *Nowellia curvifolia* | 1,14,423 bp | Sawicki et al., 2021 |
|  | *Marchantia polymorpha* | 1,21, 024 bp | Ohyama et al., 1988 |
|  | *Aneura mirabilis* | 1,08,007 bp | Yu et al., 2019 |
|  | *Haplomitrium blumei* | 1,28,728 bp | Wickett et al., 2008 |
|  | *Scapania ampliata* | 1,18,026 bp | Choi et al., 2020 |
|  | *Douinia plicata* | 1,18,797 bp | Sawicki et al., 2021 |
|  | *Cololejeunea lanciloba* | 1,08,674 bp | Sawicki et al., 2021 |
|  | *Ptilidium pulcherrimum* | 1,19,007 bp | Forrest et al., 2011 |
|  | *Apopellia* *endiviifolia* | 1,20,546 bp | Grosche et al., 2012 |
|  | *Aneura pinguis* | 1,20,698 bp | Myszczyński et al., 2017 |
|  | *Conocephalum conicum* | 1,20,963 bp | Sawicki et al., 2020 |
|  | *Conocephalum salebrosum* | 1,22,491 bp | Sawicki et al., 2020 |
|  | *Riccia fluitans* | 1, 21,999 bp | Sawicki et al., 2020 |
|  | *Sphaerocarpos texanus* | 1,21, 377 bp | Sawicki et al., 2020 |
|  | *Gymnomitrion concinnatum* | 1,20,994 bp | Myszczyński et al., 2018 |
|  | *Calypogeia muelleriana* | 1,20,137 bp | Ślipiko et al., 2020 |
|  | *Dumortiera hirsuta* | 1,22,050 bp | Kwon et al., 2019 |
|  | *Reboulia hemispherica* | 1,22,596 bp | Kwon et al., 2019 |
|  | *Pellia endiviifolia* | 1,20,546 bp | Grosche et al., 2012 |
| **S. No.** | **Bryophytes** | **Plastome Size** | **References** |
|  | *Douinia plicata* | 1,18,797 bp | Bum et al., 2020 |
|  | **Hornworts** |  |  |
|  | *Anthoceros formosae* | 1,61,162 bp | Kugita et al., 2003 |
|  | *Nothoceros aenigmaticus* | 1.53.208 bp | Villarreal et al., 2012 |
|  | *Leiosporoceros dussii* | 1,55,956 bp | Villarreal et al., 2018 |
|  | *Anthoceros agrestris* | 1,60,760 bp | Gerke et al., 2019 |
|  | **Mosses** |  |  |
|  | *Polytrichum commune* | 1,26,323 bp | Jin & Zhu, 2021 |
|  | *Physcomitrella patens* | 1,22,890 bp | Sugiura et al., 2003 |
|  | *Tortula ruralis* | 1,23,500 bp | Oliver et al., 2010 |
|  | *Sanionia uncinata* | 1,24,374 bp | Park et al., 2018 |
|  | *Pseudocrossidium replicatum* | 1,23,512 bp | Cevallos et al., 2019 |
|  | *Mnium marginatum* | 1,24,935 bp | Shi et al., 2021 |
|  | *Rhodobryum laxelimbetum* | 1,24,632 bp | Li et al., 2021 |
|  | *Climacium dendroides* | 1,24,957 bp | Han et al., 2020 |
|  | *Myuroclada maximowoczii* | 1,24,607 bp | Han et al., 2020 |
|  | *Haplocladium microphyllum* | 1,24,478 bp | Mao et al., 2020 |
|  | *Bartramia pomiformis* | 1,25,866 bp | Han et al., 2020 |
|  | Myurella julacea | 1,24,457 bp | Han et al., 2020 |
|  | *Tetraphis pellucida* | 1,27,489 bp | Bell et al., 2014 |
|  | *Syntrichia filaris* | 1,36,227 bp | Kim et al., 2019 |
|  | *Fissidens nobilis* | 1,24,962 bp | Kwon et al., 2019 |
|  | *Pohlia cruda* | 1,25,114 bp | Zhang et al., 2019 |
|  | *Leucobryum juniperoideum* | 1,24,649 bp | Min et al., 2019 |
|  | *Nyholmiella obtusifolia* | 1,22,895 bp | Sawicki, 2015 (Unpublished) |
|  | *Andreaea rupestris* | 1,35,214 bp | Jin et al., 2021 |

**Table S3:** Table showing the list of bryophytes with information about mitochondrial genome

| **S. No.** | **Bryophyte** | **Mitogenome size** | **References** |
| --- | --- | --- | --- |
|  | **Liverworts** |  |  |
|  | *Bazzania tridens* | 1,62,643 bp | Dong et al., 2019 |
|  | *Riccardia planiflora* | 1,49,371 bp | Dong et al., 2019 |
|  | *Aneura pinguis* | 1,65,603 bp | Myszczyński et al., 2017 |
|  | *Bazzania japonica* | 1,62,605 bp (Unverified) | Dong et al., 2019 |
|  | *Blasia pusila* | 1,84,923 bp (Unverified) | Dong et al., 2019 |
|  | *Calypogeia arguta* | 159,061–163,057 bp | Ślipiko et al., 2017 |
|  | *Calypogeia intergristipula* | 1,63,057 bp | Dong et al., 2019 |
|  | *Conocephalum conicum* | 1,86,148 bp (Unverified) | Dong et al., 2019 |
|  | *Dumortiera hirsuta* | 1,78,019 bp | Kwon et al., 2019 |
|  | *Douinia plicata* | 1,44,206 bp | Choi et al., 2021 |
|  | *Fossombronia cristula* | 1,79,676 bp (Unverified) | Dong et al., 2019 |
|  | *Frullania orientalis* | 1,49,360 bp (Unverified) | Dong et al., 2019 |
|  | *Gymnomitrion concinnatum* | 1,62,572 bp | Myszczyński and Sawicki (Unpublished) |
|  | *Haplomitrium mnioides* | 1,28,252 bp (Unverified) | Dong et al., 2019 |
|  | *Herbertus ramosus* | 1,16,816 bp (Unverified) | Dong et al., 2021 |
|  | *Heteroscyphus zollingeri* | 1,17,774 bp (Unverified) | Dong et al., 2021 |
|  | *Lepidozia trichodes* | 1,19,016 bp (Unverified) | Dong et al., 2021 |
|  | *Makinoa crispata* | 1,67,159 bp (Unverified) | Dong et al., 2021 |
|  | *Marchantia paleacea* | 1,86,609 bp | Oda et al., 1992 |
|  | *Marchantia polymorpha* | 1,86,196 bp | Kwon et al., 2019 |
|  | *Metacalypogeia alternifolia* | 1,19,739 bp (Unverified) | Dong et al., 2021 |
|  | *Metzgeria leptoneura* | 1,71,177 bp (Unverified) | Dong et al., 2019 |
|  | *Monosolenium tenerum* | 1,22,377 bp (Unverified) | Dong et al., 2021 |
|  | *Nowellia curvifolia* | 1,48,154- 1,48,199 bp | Sawicki et al., 2021 |
|  | *Odontoschisma grosseverrucosum* | 1,16,552 bp (Unverified) | Dong et al., 2021 |
|  | *Plagiochila subtropica* | 1,17,654 bp (Unverified) | Dong et al., 2021 |
|  | *Pleurozia purpurea* | 1,68,526 bp | Wang et al., 2009 |
|  | *Plicanthus hirtellus* | 1,18,571 bp (Unverified) | Dong et al., 2021 |
|  | *Porella plumosa* | 1,21,211 bp (Unverified) | Dong et al., 2021 |
|  | *Radula japonica* | 1,18,991 bp (Unverified) | Dong et al., 2021 |
|  | *Riccardia latifrons* | 1,18,024 bp (Unverified) | Dong et al., 2021 |
|  | *Riccia cavernosa* | 1,21,928 bp (Unverified) | Dong et al., 2021 |
|  | *Riccia fluitans* | 1,85,640 bp | Min et al., 2020 |
| **S. No.** | **Bryophyte** | **Mitogenome size** | **References** |
|  | **Liverworts** |  |  |
|  | *Scapania ornithopodioides* | 1,17,679 bp (Unverified) | Dong et al., 2021 |
|  | *Scapania ampliata* | 1,43,664 bp | Choi et al., 2021 |
|  | *Treubia lacunosa* | 1,51,983 bp | Liu et al., 2011 |
|  | *Trichocolea tomentella* | 1,19,339 bp (Unverified) | Dong et al., 2021 |
|  | *Tritomaria quinquedentata* | 1,42,510 bp | Myszczyński and Sawicki (Unpublished) |
|  | *Wiesnerella denudata* | 1,85,640 bp | Choi et al., 2021 |
|  | *Haplocladium microphyllum* | 1,24,478 bp | Mao et al., 2020 |
|  | **Hornworts** |  |  |
| 1. | Leiosporoceros dussii | 2,12,153 bp | Villarreal et al., 2018 |
| 2. | *Anthoceros angustus* | 2,42,410 bp | Dong et al., 2018 |
| 3. | *Phaeoceros laevis* | 2,09,482 bp | Xue et al., 2010 |
| 4. | *Megaceros aenigmaticus* | 1,84,908 bp | Li et al., 2009 |
|  | **Mosses** |  |  |
|  | *Physcomitrella patens* | 1,05,340 bp | Terasawa et al., 2007 |
|  | *Mielichhoferia elongata* | 1,00,342 bp | Goryunov et al., 2018 |
|  | *Climacium dendroides* | 1,04,860 bp | Choi et al., 2020 |
|  | *Syntrichia filaris* | 1,06,343 bp | Yoon et al., 2015 |
| 5. | *Polytrichum commune* | 1,14,831 bp | Goryunov et al., 2021 |
| 6. | *Chorisodontium aciphyllum* | 1,05,766 bp | Byun et al., 2019 |
| 7. | *Myurella julacea* | 104,979 bp | Jung et al., 2021 |
| 8. | *Orthotrichum diaphanum* | 1,04,744 bp | Vigalondo et al., 2016 |
| 9. | Orthotrichum *macrocephalum* | 1,04,624 bp | Vigalondo et al., 2016 |
| 10. | *Orthotrichum speciousum* | 104,747 bp | Sawicki et al., 2014 |
| 11. | *Orthotrichum rogeri* | 106,634 bp | Jakub et al., 2015 |
| 12. | *Bartramia pomiformis* | 106,155 bp | Park et al., 2020 |
| 13. | *Brachythecium rivulare* | 104,460 bp | Goryunov et al., 2017 |
| 14. | *Oxystegus tenuirostris* | 105 001 bp | Alonso et al., 2015 |
| 15. | *Pseudocrossidium replicatum* | 105,495 bp | Cevallos et al., 2020 |
| 16. | Anomodon rugelii | 104,239 bp | Liu et al., 2011 |
| **S. No.** | **Bryophyte** | **Mitogenome size** | **References** |
|  | **Mosses** |  |  |
| 17. | *Myuroclada maximowiczii* | 104,216 bp | Han et al., 2021 |
| 18. | *Codriophorus laevigatus* | 106,809 bp | Szczecińska et al., 2016 |
| 19. | *Anomodon attenuatus* | 125,320 bp | Chen et al., 2020 |
| 20. | Tetraplodon fuegianus | 123,670–123,672 bp | Lewis et al., 2016 |
| 21. | *Bartramia patens* | 106,827 bp | Cho et al., 2019 |
| 22. | *Niphotrichum ericoides* | 106,727 bp | Szczecińska et al., 2015 |
| 23. | *Pohlia nutans* | 99864 bp | Cao et al., 2020 |
| 24. | *Bartramia pomiformis* | 1,25,866 bp | Han et al., 2020 |
| 25. | *Myurella julacea* | 1,24,457 bp | Han et al., 2020 |
